# Assembling the perfect bacterial genome using Oxford Nanopore and Illumina sequencing

**DOI:** 10.1371/journal.pcbi.1010905

**Published:** 2023-03-02

**Authors:** Ryan R. Wick, Louise M. Judd, Kathryn E. Holt

**Affiliations:** 1 Department of Infectious Diseases, Central Clinical School, Monash University, Melbourne, Australia; 2 Department of Microbiology and Immunology, University of Melbourne at the Peter Doherty Institute for Infection and Immunity, Melbourne, Australia; 3 Department of Infection Biology, London School of Hygiene & Tropical Medicine, London, United Kingdom; McGill University, CANADA

## Abstract

A perfect bacterial genome assembly is one where the assembled sequence is an exact match for the organism’s genome—each replicon sequence is complete and contains no errors. While this has been difficult to achieve in the past, improvements in long-read sequencing, assemblers, and polishers have brought perfect assemblies within reach. Here, we describe our recommended approach for assembling a bacterial genome to perfection using a combination of Oxford Nanopore Technologies long reads and Illumina short reads: Trycycler long-read assembly, Medaka long-read polishing, Polypolish short-read polishing, followed by other short-read polishing tools and manual curation. We also discuss potential pitfalls one might encounter when assembling challenging genomes, and we provide an online tutorial with sample data (github.com/rrwick/perfect-bacterial-genome-tutorial).

## Introduction

Compared to eukaryotes, which have complex genomes often exceeding 1 billion base pairs (bp) in length, prokaryote genomes are small, typically containing a single circular chromosome a few million bp in length and often small extrachromosomal plasmids [1]. In many genomic applications, it would be most useful to know the bacterial genome sequence in its entirety, i.e., the full sequence of nucleotides for each piece of DNA in the cell. However, DNA sequencers work by fragmenting the genome and sequencing the fragments, producing reads: randomly ordered small pieces of the genome [2]. Reads are imperfect, with the frequency and type of errors depending on the platform. To ensure that every part of the genome is sequenced multiple times (i.e., none of the genome is missed), it is necessary to produce reads that total to many times the genome size. There is thus a disconnect between what sequencers provide (small, imperfect, overlapping sequences) and what we want (a complete, error-free genome).

The solution to this problem is de novo assembly: the computational process of reconstructing a genome from sequencing reads. There are two broad goals to consider with genome assembly: accuracy and completeness. Accuracy refers to the number of errors present in the assembled sequences (contigs). Such errors can be small in scale (e.g., an incorrect base) or larger in scale (e.g., the addition, removal, or inversion of hundreds of bases). Completeness refers to the length of the contigs relative to the corresponding genomic sequence, i.e., how fragmented the assembly is. Longer contigs are better, ideally each contig representing an entire replicon in the genome. We define a “perfect” assembly as one with 100% accuracy (no errors) and maximal completeness (one contig per replicon and no additional contigs).

Many downstream analyses do not require high-quality assemblies, e.g., one can identify the species of a genome or the presence/absence of a gene using a low-quality draft assembly [3]. There are, however, tasks that require extreme accuracy, e.g., estimating mutation rates and inferring transmission chains, where even a small number of errors can have consequences. Perfect assemblies offer no limits on their downstream uses, making “is my assembly good enough?” an irrelevant question. In the absence of assembly errors, many analyses that involve interrogating reads directly (using computationally intensive approaches, e.g., variant calling) could be replaced by simpler assembly-based alternatives such as whole-genome alignment.

**Fig 1:**
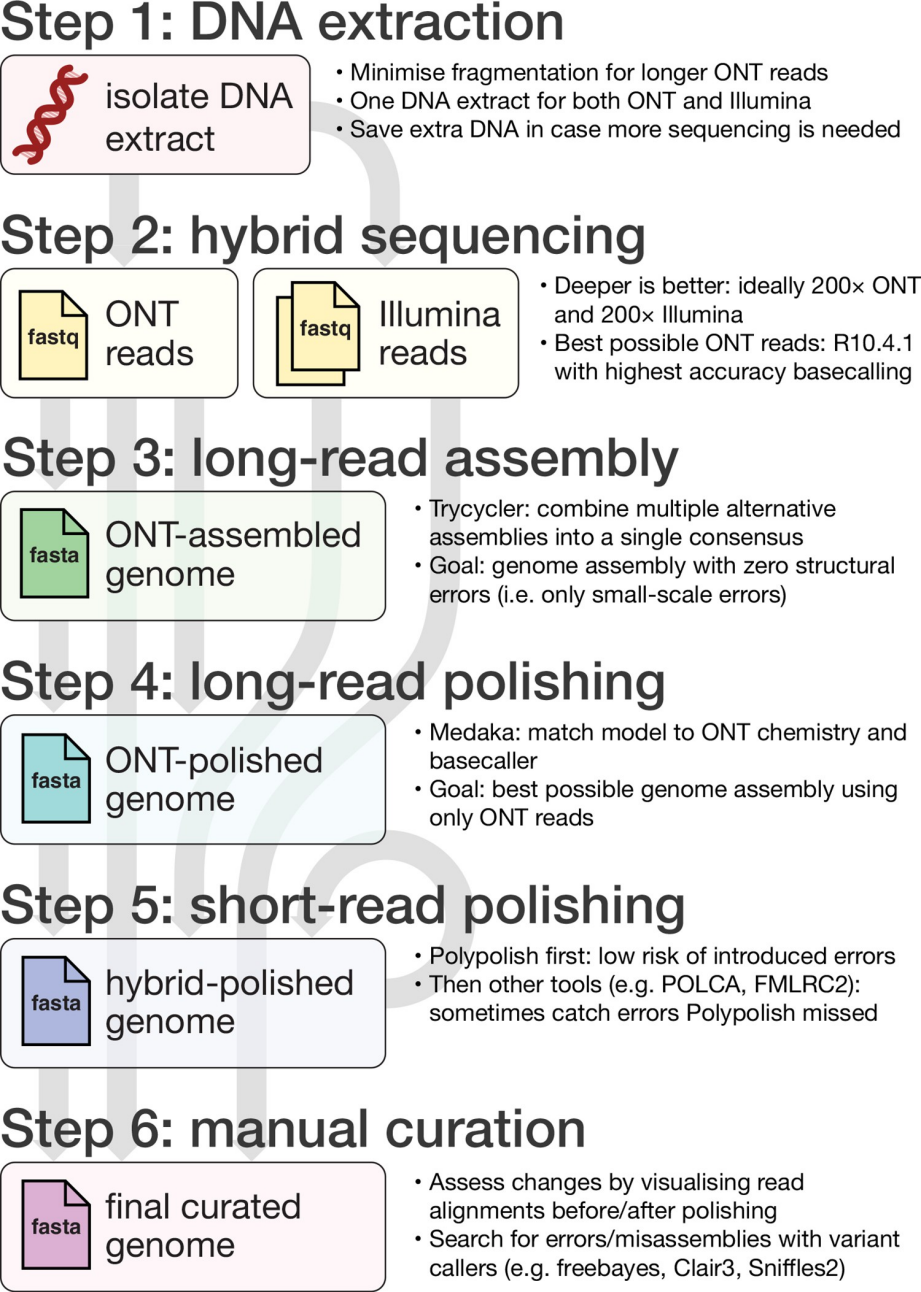
Illustrated overview of our recommended approach to perfect bacterial whole-genome assembly.

Here, we describe a current approach for producing a bacterial genome assembly with the goal of perfection using a combination of Oxford Nanopore Technologies (ONT) long reads and Illumina short reads (**Fig 1**). While PacBio HiFi reads have low error rates and can produce very accurate assemblies of bacterial genomes [4], we chose to focus on ONT and Illumina platforms for their availability and widespread adoption in microbial genomics. Older hybrid assembly methods have used a short-read-first approach (building a short-read assembly graph and then scaffolding with long reads) [5], but improvements in the yield and accuracy of long-read sequencing now mean that long-read-first hybrid assembly (making a long-read-only assembly and then polishing with short reads) can produce more accurate results [6], and that is the approach we use here. We also provide an online tutorial (github.com/rrwick/perfect-bacterial-genome-tutorial) with sample data (hybrid sequencing of *Staphylococcus aureus* strain JKD6159; [7]) so readers can try this method for themselves.

## Step 1: DNA extraction

DNA should be extracted from a culture grown from a single bacterial colony to minimise the chance of genomic heterogeneity (see **Pitfalls**). While the best method for extracting DNA can vary by organism, one should aim to maximise purity and molecular weight. High purity will allow for better ONT yields, as chemical and biological impurities can damage or clog nanopores, shortening the life of flow cells [8]. High molecular weight will produce longer ONT reads, so one should avoid vortexing, minimise handling/pipetting, and minimise freeze–thaw cycles to reduce shearing of DNA molecules [9]. Extraction methods for most bacteria should incorporate cell lysis by enzymatic digestion, using lysozyme (Sigma Aldrich, L6876) followed by proteinase K digestion (as provided in DNA extraction kits). This method is suitable for most gram-negative and gram-positive bacteria, but optimisation with additional enzymes may be required for difficult-to-lyse bacteria. Magnetic bead-based DNA extraction is recommended to reduce DNA shearing and maximise throughput. Recommended kits (in order of preference) are GenFind V3 (Beckman Coulter, C34881) and MagAttract HMW DNA (Qiagen, 67563). For bacterial isolates that are difficult to lyse enzymatically, bead-beating can be used, but ONT read length may be compromised.

If culturing and DNA extraction is conducted multiple times (e.g., once for ONT sequencing and again for Illumina sequencing), there is the risk of genomic differences between the DNA samples [10]. This can lead to difficulties during polishing, so we recommend using a single DNA extract for all sequencing runs. It may also be prudent to freeze additional DNA or bacterial pellets in case further sequencing is later required.

## Step 2: Sequencing

### Long-read ONT sequencing

One key consideration for ONT sequencing is depth, defined as the total number of sequenced bases divided by the genome size, i.e., the mean number of reads covering each part of the genome. High read depth aids both assembly (allowing for more independent read sets in Trycycler; see **Step 3**) and polishing (yielding higher accuracy; see **Step 4**). When aiming for a perfect assembly, consider 100× depth to be a minimum, with 200× being ideal. Depths above 200× are better but will give diminishing returns. Using a single ONT flow cell for one bacterial isolate may provide excessive depth, so multiplexing is common in microbial genomics. For example, with a 5-Mbp genome size, a target depth of 200× and an expected yield of 10 Gbp, one could sequence 10 isolates on a single MinION/GridION flow cell. Multiplexing is not a problem for assembly, though barcode leakage should be considered (see **Pitfalls**).

Another consideration is length: How long must the ONT reads be? N50 length, the length-weighted median, is a commonly used metric [11]. To ensure a complete assembly, the read set should have an N50 length greater than the longest repeat sequence. For many bacterial genomes, this is the rRNA operon, which is approximately 5 kbp and usually present in multiple copies [12], making an ONT read N50 of approximately 20 kbp a good target. In rare cases where the genome has an unusually long repeat (see **Pitfalls**), ultralong DNA extraction protocols may be necessary [13].

ONT library preparation and chemistry are also important factors. Both ligation-based and rapid preparations are appropriate for bacterial whole-genome sequencing, though ligation-based preparations can favour sequencing yield while rapid preparations can favour read length [13,14]. ONT currently offers MinION/GridION flow cells with two different pores: R9.4.1 (released in 2017) and R10.4.1 (released in 2022). The pores used in R10.4.1 flow cells are longer, improving homopolymer resolution and consensus accuracy, making them the better choice for assembly [15].

Basecalling, the computational process of translating the sequencer’s raw signals into nucleotide sequences, is under constant development, so users should opt for the most recent version of ONT’s recommended basecaller and use its highest accuracy model. If users do not have an ONT sequencer with a GPU (e.g., a GridION), then access to a GPU will be required to perform basecalling. Retaining the raw reads (FAST5 or POD5 format) is recommended, as future basecallers may allow for rebasecalling with increased accuracy.

After basecalling, QC filtering can improve the quality of the ONT reads. We recommend using Filtlong [16] to remove the worst reads (short length and low accuracy) with --keep_percent 90. If the read set has a poor N50 but is very deep, then removing short reads (e.g., <5 kbp) can help with assembly, though this may compromise small plasmid recovery (see **Pitfalls**). If adapters were not trimmed by the basecaller, they can be trimmed using an external program such as SNIKT [17], though we have previously found that untrimmed adapters have little effect on assembly [18].

### Short-read Illumina sequencing

Since Illumina reads will only be used for final polishing (see **Step 5**), they carry less importance than ONT reads. While accuracy can vary between current Illumina platforms [19], most produce similar data (e.g., 150-bp paired-end reads, less than 1% errors) and will function equally well in polishing algorithms, with instrument choice driven by cost and multiplexing needs. Nextera XT library preparations result in variable read depth (i.e., some regions of the genome may have low depth), so Illumina DNA Prep (a.k.a. Nextera DNA Flex) and TruSeq are preferable [20]. If Nextera XT is used, aim for a high mean depth (e.g., 300×) to compensate for depth variation; otherwise, 100× should be sufficient. For highly repetitive genomes, mate-pair preparations may improve short-read polishing performance (see **Pitfalls**). After Illumina reads are produced, we recommend using a QC tool such as fastp [21] to remove low-quality bases and adapter sequences.

## Step 3: Long-read assembly

The goal of long-read assembly is to produce complete sequences with no structural errors, i.e., the only errors in the assembly should be small scale, e.g., single-bp substitutions, insertions, or deletions. This is because later polishing steps can repair small-scale errors but may not be able to fix larger structural errors.

Several long-read assemblers have been developed that are suitable for bacterial genomes, including Canu [22], Flye [23], NECAT [24], NextDenovo [25], and Raven [26], each of which uses different methods and thus has advantages/disadvantages. Regardless of the assembler used, most long-read bacterial genome assemblies contain avoidable errors, and given the same read set, different assemblers are likely to produce assemblies with different errors [18]. Trycycler exploits this fact by building a consensus from multiple alternative assemblies of the same genome, allowing it to avoid structural errors, remove spurious contigs, and ensure that circular sequences have no missing/duplicated bases at their ends [6]. We therefore recommend using Trycycler to produce long-read bacterial genome assemblies. However, note that Trycycler is not an automated tool—it requires human judgement and interaction.

## Step 4: Long-read polishing

This step aims to fix as many remaining errors as possible using only long reads. We recommend using Medaka [27], which we have found to produce more accurate results than Nanopolish [28,29]. Medaka uses a neural network and comes with trained models that correspond to specific combinations of ONT chemistry and basecaller, so one should choose the Medaka model which most closely matches their ONT reads. Alternatively, long-read variant callers such as Clair3 [30] can be used as polishers by applying the called variants to the assembly.

Long-read polishing is done before short-read polishing because it is less influenced by genomic repeats. A “repeat” in this context is a sequence that causes reads to align to multiple and/or incorrect positions of the genome. For example, some 150-bp short reads will be contained within the rRNA operon and will therefore align to multiple places, making the operon a repeat and impairing the ability of polishers to repair errors. With 20-kbp long reads, however, all can span the rRNA operon and therefore align uniquely, so the operon is not a repeat, ensuring that polishing changes occur in the correct instance of the operon.

Long-read polishing usually improves assembly accuracy, but a drop in accuracy is sometimes possible. It can therefore be unclear at this step whether the unpolished assembly, Medaka-polished assembly or some alternative (e.g., Clair3-polished) is best. ALE is a tool that quantifies the concordance between an assembly and a short-read set [31], allowing one to assess the relative accuracy of different assemblies. We therefore recommend using ALE to guide the decision regarding which version of the assembly should progress to the next step (short-read polishing).

## Step 5: Short-read polishing

The previous steps have generated a long-read-only assembly of maximal accuracy, likely approximately Q50 (one error per 100 kbp) if R10.4.1 ONT reads were used. The final step is to repair any remaining errors with short reads. For example, long homopolymers can be difficult for ONT sequencing to resolve [15], but Illumina sequencing does not suffer from this problem [13,32], so homopolymer-length errors which persist after long-read polishing, can be fixed by short-read polishing.

Our tool Polypolish [33] was designed with two goals in mind. The first was to use all-per-read alignments to overcome some of the constraints imposed by repeats. The second was to be very conservative, i.e., to minimise the chance of introducing errors during polishing. Polypolish only makes changes that are unambiguously supported by the read alignments, so when there are multiple possibilities at a locus (e.g., a base could be A or C with some alignments supporting each), Polypolish will not change the sequence. For this reason, we recommend running Polypolish before any other short-read polisher.

Due to its conservativeness, Polypolish may miss errors that other short-read polishers can fix, e.g., in regions of low Illumina depth. We therefore recommend trying other short-read polishers, including POLCA [34] (due to its low rate of introduced errors) and FMLRC2 [35] (due to its ability to fix errors other polishers cannot). However, other polishers can introduce new errors [33], which is unacceptable when aiming for perfection, so any changes made will need to be manually assessed.

## Step 6: Manual curation

To assess a polishing change, we recommend viewing the read alignments before and after the change using a tool such as the Integrated Genomics Viewer (IGV) [36]. This can clarify whether the change fixed an error (in which case it should be retained) or introduced an error (in which case it should be rejected) [37]. See the accompanying online tutorial (github.com/rrwick/perfect-bacterial-genome-tutorial) for commands, supporting scripts, and examples.

Tools such as freebayes [38] (short-read small variant caller), Clair3 [30] (long-read small variant caller), and Sniffles2 [39] (long-read structural variant caller) can be used to look for errors, misassemblies, and heterogeneity (see **Pitfalls**) in the final assembly. Any anomalies found can then be investigated using IGV. Other advanced methods for assembly interrogation have been developed in the field of human genomics [40,41], some of which may also be applicable to bacterial genomes.

During curation, the quality of an assembly can be quantified using a number of tools, including ALE (see **Step 4**), BUSCO [42], QUAST [43], and IDEEL [44]. While none of these tools can reliably distinguish perfect assemblies from assemblies with errors, they can provide relative metrics to weigh alternative assemblies against each other.

## Automation

The above-described method requires human judgement and interaction, particularly during Trycycler and manual curation, allowing users to catch unexpected results, ensuring that poor data do not proceed to the next step. This method is appropriate where accuracy is paramount (e.g., reference genome assembly), but it cannot be run in an automated manner (e.g., with Nextflow [45]) and is thus not suitable for high-throughput assembly.

If automation is required, changes in the workflow are needed. Flye [23] is less likely than other long-read assemblers to produce large-scale errors, which downstream polishers may not be able to fix [18], making it a good replacement for Trycycler. Before polishing with Medaka, circular Flye contigs should be “rotated” to a consistent starting sequence (e.g., *dnaA*; [46]) or random starting sequence. This will serve to move any duplicated/missing bases at the start/end of circular contigs to the middle of the sequence where polishing tools can repair the error. For short-read polishing, we recommend Polypolish followed by POLCA, as these tools are the least likely to introduce errors [33].

Users should not assume that automated assemblies are error free. In particular, structural errors (fragmented replicons, doubled plasmids, etc.) are possible, as these are what Trycycler aims to avoid.

## Pitfalls

**Fig 2:**
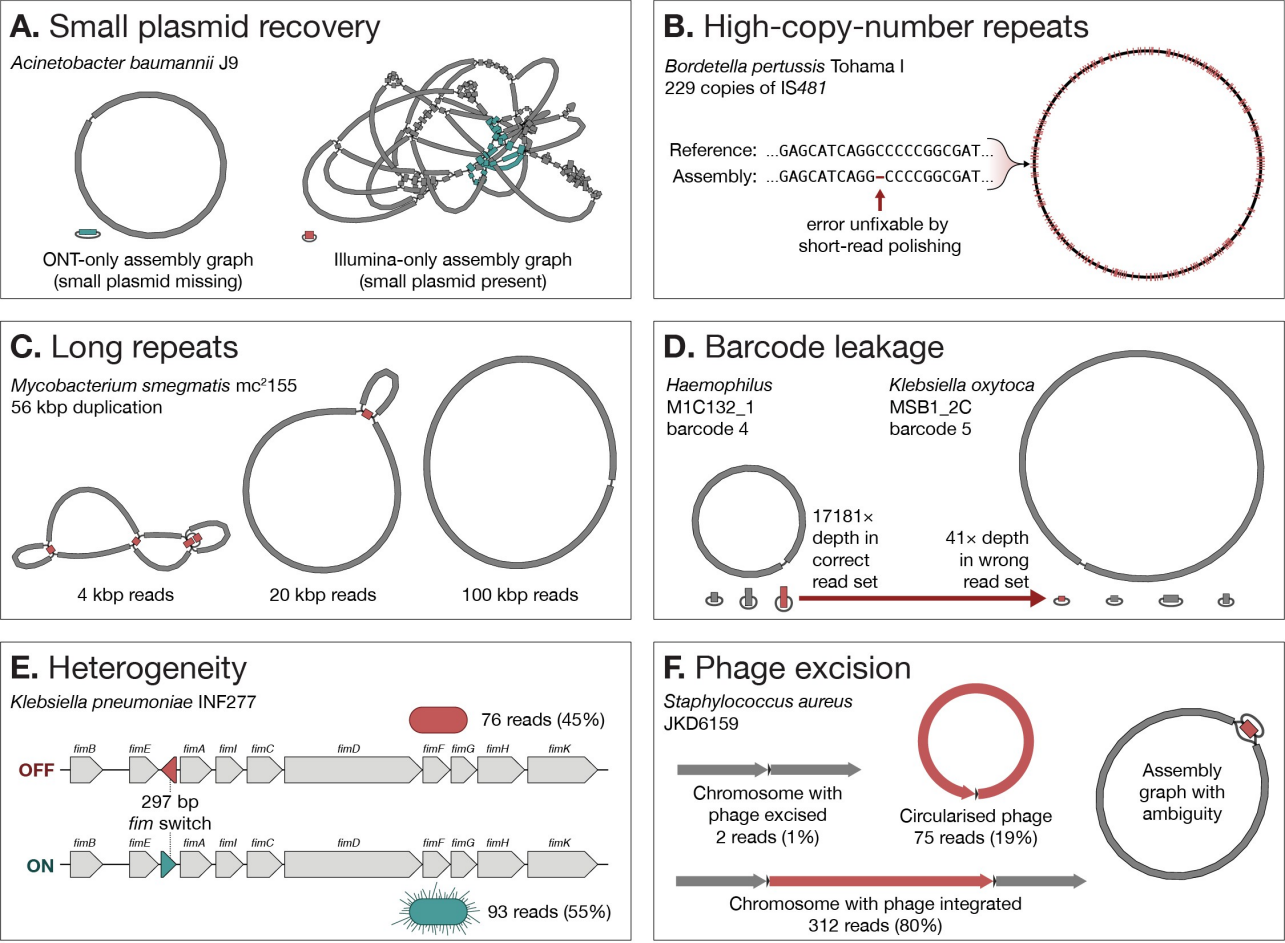
Examples of pitfalls in bacterial genome assembly and polishing. (**A)** *A*. *baumannii* J9 [47] contains one large 145-kbp plasmid (blue) and one small 6-kbp plasmid (red). The small plasmid is missing from an ONT-only assembly of this genome (left). However, it assembled completely in an Illumina-only assembly (right), enabling its recovery. (**B)** IS*481* is a repeat in the *B*. *pertussis* Tohama I genome [53]. Due to its high copy-number, some errors in this repeat are not fixable using paired-end Illumina reads and short-read polishers. (**C)** If a genome contains a very long repeat, as is the case with *M*. *smegmatis* mc^2^155 [51], typical ONT read lengths of approximately 20 kbp may not be sufficient for complete assembly. (**D)** As occurred with *Haemophilus* M1C132_1 and *K*. *oxytoca* MSB1_2C [6], read demultiplexing errors can cause a deeply sequenced replicon in one genome (left) to erroneously appear in the assembly of another genome from the same sequencing run (right). (**E)** ONT sequencing of *K*. *pneumoniae* INF277 [54] contained a near-50:50 mixture of *fim* switch orientations, causing problems during long-read and short-read polishing. (**F)** *S*. *aureus* JKD6159 [7] read sets contained structural heterogeneity around the ΦSa3 bacteriophage sequence (left), causing an incomplete Flye assembly graph (right).

Small plasmids (<20 kbp) can be underrepresented in ONT read sets, due to either ligation preparations (where circular sequences fail to acquire adapters; [47]) or overly aggressive QC (e.g., discarding all reads <10 kbp). This can be avoided by using rapid preparations and less stringent QC (e.g., only discarding reads <1 kbp). Alternatively, small plasmids can be recovered from an Illumina-only or short-read-first-hybrid assembly graph (e.g., from Unicycler; [5]) where they usually appear as circular contigs separate from the rest of the genome (**Fig 2A**).

Some bacterial taxa have undergone proliferation of insertion sequence elements in their evolution, resulting in genomes with hundreds of 1- to 2-kbp repeats [48,49]. Perfect assembly of such genomes can be challenging because short-read polishers struggle to repair errors in high copy-number repeats (**Fig 2B**). For this reason, it is crucial to maximise ONT-only accuracy (using high ONT depth, R10.4.1 pores, basecalling with the highest accuracy model, and Medaka polishing) to minimise the number of errors left for short-read polishing to fix. Additionally, mate-pair Illumina sequencing may enable Polypolish to fix errors within repeat sequences by reducing the number of ambiguous short-read alignments [50].

While the approximately 5-kbp rRNA operon is the longest repeat in many bacterial genomes, longer repeats are possible. For example, *Mycobacterium smegmatis* mc^2^155 contains a 56-kbp duplication in its chromosome [51]. In such cases, typical ONT read lengths (approximately 20 kbp) can be insufficient for assembly and ultralong reads (approximately 100 kbp) are needed (**Fig 2C**).

In multiplexed sequencing runs, some reads from one barcode can “leak” into another, resulting in low-level contamination [52]. This can originate during library preparation (e.g., barcodes failing to ligate until after sample pooling) or during computational steps (e.g., basecalling errors in a barcode sequence causing incorrect demultiplexing). When a sequence in one barcode is very high depth, it may appear in other barcodes at sufficient depths to be assembled. This most often occurs with high copy-number plasmids (**Fig 2D**), so when multiple genome assemblies from the same sequencing run contain identical plasmids, cross-barcode contamination should be considered as a possible cause.

Heterogeneity occurs when there is not a single underlying genome but rather a mixture of two or more alternatives. This can occur at small scales (e.g., a mixture of different bases at a locus) or large scales (e.g., a mixture of structural configurations). The concept of assembly perfection can be unclear in the presence of heterogeneity, but for simplicity, we will consider a perfect assembly of a heterogenous genome to contain the most common sequence at each variable locus. When heterogeneity occurs at a low level (e.g., 95% of the reads support one sequence and 5% another), it does not typically cause problems as assemblers/polishers will use the more common alternative. However, balanced heterogeneity (e.g., a near-50:50 mixture) can cause misassemblies and polishing mistakes. The phase variation of the *fim* switch is one cause of heterogeneity in *Enterobacteriaceae* [55] (**Fig 2E**). Another common example occurs with bacteriophages, which can integrate into and excise from bacterial chromosomes [56] (**Fig 2F**). Heterogeneity can be identified by incomplete assembly graphs and dense clusters of changes made by a polisher. It may then be necessary to manually exclude reads that support one alternative, allowing the other alternative to assemble/polish cleanly.

## Conclusions

In contrast to short-read-first hybrid assembly approaches of the past (e.g., Unicycler), our recommended method follows a long-read-first paradigm. Due to their improved handling of repeats, long reads form a solid assembly foundation, with short reads only used for final polishing. If the long-read assembly is sufficiently accurate (ideally Q50 or greater, i.e., less than one error per 100 kbp), then short-read polishing can often repair all remaining errors, making perfect genome assemblies achievable. However, it is not easy to establish a ground truth genome sequence, so when assembly accuracy is critical, we recommend performing multiple alternative assemblies that vary in data/methods: sequencing platforms, assemblers in the Trycycler pipeline, read QC thresholds, short-read polishing tools, etc. When alternative data/methods produce identical assemblies, this builds confidence in their correctness. When alternative assemblies are not identical, further investigation (e.g., visualising read alignments in IGV) is warranted.

While perfect bacterial genome assemblies are now possible, they are not yet simple to produce. The future will undoubtedly bring improvements to ONT chemistry, basecallers, and polishers, but whether these will be sufficient for perfect ONT-only assemblies (negating the need for Illumina reads) remains to be seen. Further software developments are needed to remove the human-interaction elements, enabling perfect assemblies from a fully automated pipeline, even in complicated cases (e.g., genomes with heterogeneity). The ultimate goal is a future where genomes can be assembled to perfection with enough ease and reliability that it is taken for granted.
